# A systematic review of the determinants of implementation of a locomotor training program using a powered exoskeleton for individuals with a spinal cord injury

**DOI:** 10.1177/02692155231164092

**Published:** 2023-04-10

**Authors:** Caroline Charette, Julien Déry, Andreanne K Blanchette, Céline Faure, François Routhier, Laurent J Bouyer, Marie-Eve Lamontagne

**Affiliations:** 1 Department of Rehabilitation, 4440 Université Laval, Quebec City, Canada; 2 560498 Centre for Interdisciplinary Research in Rehabilitation and Social Integration, 113463 Centre intégré universitaire de santé et de services sociaux de la Capitale-Nationale du Québec, Quebec City, Canada; 3 Thematic Center for Research in Neuroscience, Quebec City, Canada

**Keywords:** Robotics, perceptions, gait, barriers, facilitators

## Abstract

**Background:**

Wearable powered exoskeletons represent a promising rehabilitation tool for locomotor training in various populations, including in individuals with a spinal cord injury. The lack of clear evidence on how to implement a locomotor powered exoskeleton training program raises many challenges for patients, clinicians and organizations.

**Objective:**

To report determinants of implementation in clinical practice of an overground powered exoskeleton locomotor training program for persons with a spinal cord injury.

**Data sources:**

Medline, CINAHL, Web of Science.

**Study selection:**

Studies were included if they documented determinants of implementation of an overground powered exoskeleton locomotor training program for individuals with spinal cord injury.

**Data extraction:**

Eligible studies were identified by two independent reviewers. Data were extracted by one reviewer, based on constructs of the Consolidated Framework for Implementation Research, and validated by a second reviewer.

**Results:**

Sixty-three articles were included. 49.4% of all determinants identified were related to the intervention characteristics, 29.6% to the individuals’ characteristic and 13.5% to the inner setting. Recurrent barriers identified were the high prevalence of adverse events (e.g., skin issues, falls) and device malfunctions. Adequate training for clinicians, time and resource available, as well as discussion about patients’ expectations were identified as facilitators.

**Conclusions:**

Powered exoskeleton training is a complex intervention. The limited information on the context and the implementation process domains may represent a barrier to a successful transition from knowledge to action.

## Background

In Canada, over 86,000 people live with a spinal cord injury,^1^ which may lead to sensorimotor disorders, autonomic dysfunctions, and walking limitations. Irrespective of age, injury severity, or time since injury, recovery of walking is a high priority for them.^2^ However, walking limitations often remain after rehabilitation that can negatively impact participation in daily activities and quality of life.^3,4^

Wearable powered exoskeletons represent a promising rehabilitation tool for standing and locomotor training that may complement conventional locomotor training after a spinal cord injury by enabling more repetition and intensive exercise.^5,6^ Systematic reviews have shown functional and health benefits associated with overground powered exoskeleton locomotor training for individuals with spinal cord injury, such as improvements in walking speed and endurance, standing balance, lower extremity function, bowel function, as well as reduction in neuropathic pain and spasm intensity.^7–11^

Integrating the use of powered exoskeletons as part of rehabilitation practice is not a simple matter, however. In a study on robotic technologies for upper-limb impairment rehabilitation, the authors concluded that the lack of clear evidence that takes into consideration all relevant dimensions of implementation is one of the most important barriers to a wider adoption of robotic technologies.^12^ Research on adoption and implementation of robotics in stroke rehabilitation demonstrated that the clinical uptake of these technologies is relatively low.^13,14^ Numerous barriers have been identified, such as the lack of training combined with little or no allocated time for training for clinician, or the fact that using the technology was perceived as being too time-consuming by clinicians.^14^ Thus, from the early stages of development to its implementation in real-life settings, it is crucial to take into consideration the perception of potential users, such as clinicians and patients, toward a technology to ensure it adequately matches their needs and requirements.^15,16^ Moreover, therapists highlighted the pressing need for guidance regarding the integration of exoskeletons into existing rehabilitation services.^17^ Therefore, identifying and addressing barriers early in the development of this intervention is essential for the research to clinical knowledge translation^18^ and facilitates effective clinical adoption of wearable powered exoskeletons. The purpose of this knowledge synthesis is therefore to systematically report the potential determinants of implementation of an overground powered exoskeleton locomotor training program for individuals with a spinal cord injury.

## Methods

The knowledge synthesis protocol has been registered within the PROSPERO database (registration number: CRD42021239327) and is being reported in accordance with the Preferred Reporting Items for Systematic Reviews and Meta-Analyses (2020) statement.^19^ A review protocol was not published prior to this systematic review.

A literature search was conducted, in September 2020, using Medline, CINAHL, and Web of science databases. An update of the review search was done in June 2022. A search strategy combining keywords and indexed vocabulary related to the following key concepts: [1] exoskeleton, [2] locomotion or gait, [3] spinal cord injury, and [4] implementation determinants (facilitators and barriers to the use or to the implementation of powered exoskeletons) was used. The search strategy, which was developed by the research team and a professional librarian, was adjusted for each database (detailed search strategy presented in Appendix 1). The reference lists of selected studies and related systematic reviews(8, 9) were also searched for additional references.

Articles were included if: [1] they were peer-reviewed empirical studies, including qualitative, quantitative, or mixed methods; [2] they were published in English or in French; [3] they referred to an overground locomotor training program using a wearable powered multi-joint lower limb exoskeleton in clinical practice; [4] the powered exoskeleton locomotor training targeted adults (16 years old and older) with a subacute or chronic spinal cord injury (no specificity regarding the spinal cord injury level); and if [5] determinants of implementation of a powered exoskeleton locomotor training program were reported. Publications were excluded if: [1] they consisted of conference proceedings, abstracts, commentaries, letters, book chapters, animal studies, theses, reviews, meta-analyses, and proof-of-concepts; [2] the locomotor training involved less than three sessions, or consisted of fitting or parameter testing only; [3] the studied population included participants with various neurological conditions (or pathologies) and the perceptions of individuals with a spinal cord injury could not be extracted; and if [4] the powered exoskeleton was considered or used for personal mobility purposes at-home or in the community, rather than for rehabilitation training. As this technology is relatively recent, there were no restrictions on the year of publication.

The main outcome of this systematic review was the determinants of implementation, which are often identified as barriers and facilitators. These determinants may have been reported by different stakeholders (e.g., clinicians, patients, administrators) or by researchers who conducted the empirical studies.

After identifying studies through databases and eliminating duplicates, titles and abstracts were screened by two independent reviewers (CC and JD). Relevant full texts were then retrieved and independently assessed for eligibility by the same reviewers. Disagreements between reviewers were discussed, and a third reviewer (AKB) was consulted for final decision, when needed.

All the selected studies that met eligibility criteria were reviewed and information related to study characteristics, such as author, year of publication, design (i.e., quantitative, qualitative, or mixed methods), participant characteristics and type of exoskeleton (if applicable) was extracted by one reviewer (JD). For the extraction of determinants, one reviewer (JD) performed the initial data extraction using Dedoose Software (www.dedoose.com). Then, a second reviewer (CC) read the complete articles to validate this initial extraction and identified any additional determinants. Modification or addition to the first extraction was discussed between the two reviewers. The information extracted was validated by a third reviewer (MEL) in case of any disagreements.

To ensure that all relevant dimensions of implementation were taken into consideration, the Consolidated Framework for Implementation Research (20) was used to guide the data extraction process and to synthesize findings. This framework aims to offer a consistent, standardized taxonomy, terminology and definition of facilitators and barriers involved in health care implementation research, and includes five major domains, namely [1] *intervention characteristics*, [2] *outer setting*, [3] *inner setting*, [4] *characteristics of the individuals* involved, and [5] *process of implementation* (www.cfirguide.org).^20^

A narrative synthesis method was then used to describe the potential determinants of implementation and use of an overground powered exoskeleton locomotor training program for individuals with a spinal cord injury.

## Results

**Figure 1.**
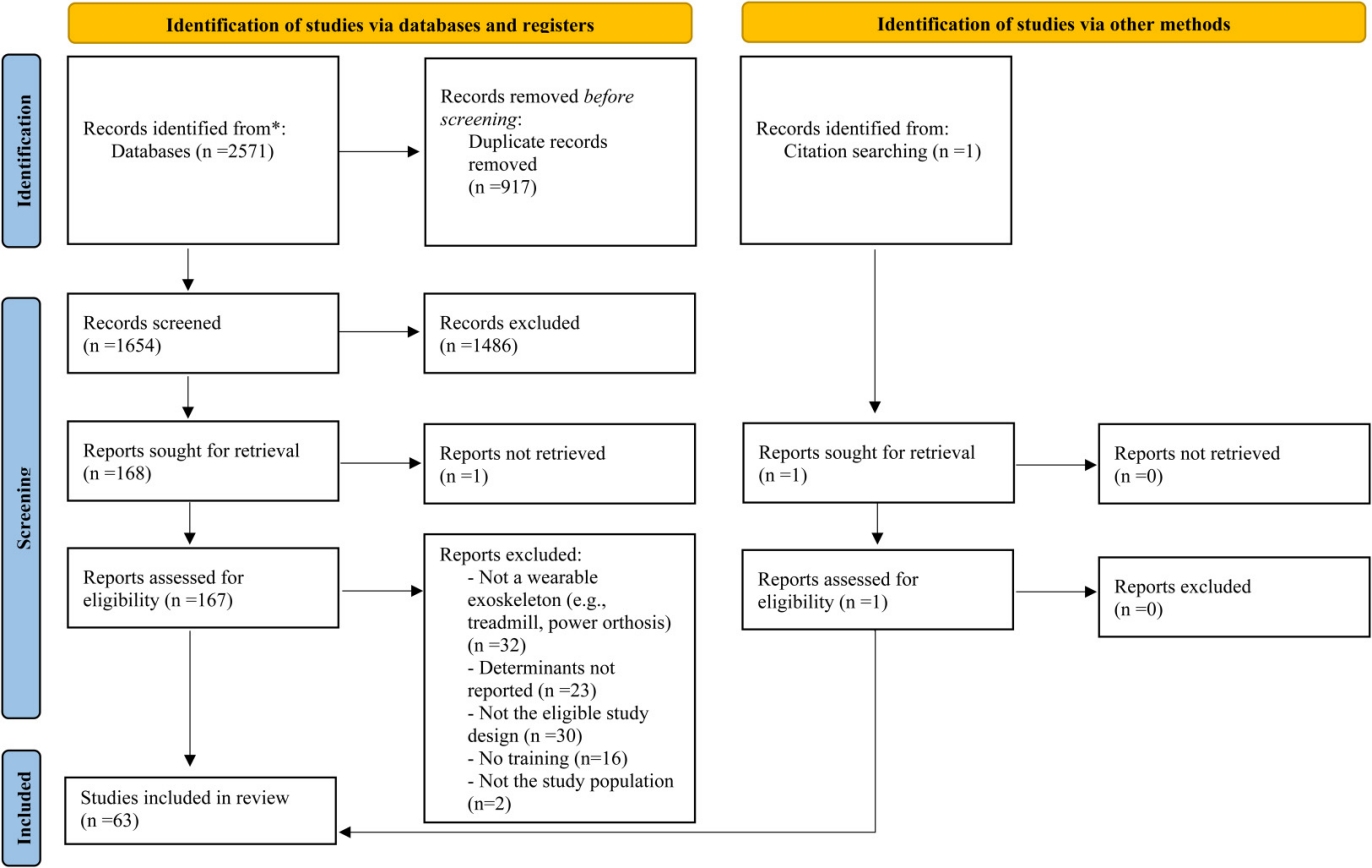
Article selection process: PRISMA flow diagram from Page et al. (2021).^19^

The database literature searches result in the identification of 2571 references and one additional record was identified through review of reference lists (for a total of 2572 references). Of this total, 1654 articles remained after removing duplicates. After the screening of titles and abstracts, 169 full text articles were assessed for eligibility and 105 did not meet the selection criteria. Figure 1 presents the PRISMA flow diagram for the inclusion of 63 relevant papers.^5,6,17,21–80^ The update of the literature search added 20 studies.

All selected studies were published between 2012 and 2022. Most studies used quantitative research methods (51/63), while the others used qualitative research (9/63) and mixed methods (3/63). The primary objectives of these studies were related to the assessment of feasibility, efficacy, or effectiveness of an overground powered exoskeleton gait training in individuals with a spinal cord injury. Twelve studies were designed as retrospective evaluation of barriers and facilitators to the development and implementation of powered exoskeletons gait training for individuals with a spinal cord injury, which were documented through surveys, focus groups and/or individual interviews.^17,21–31^ Among these studies, 7 explored the perspective of persons with a spinal cord injury,^21,23–25,28–30^ while 7 documented clinicians’ perspective.^17,22,26–29,31^ Different models of wearable powered exoskeleton were used in the included studies (Ekso, n = 31; ReWalk, n = 20; Indego, n = 5; WPAL, n = 4; and others, n = 9). For a more detailed overview of the included articles, see the supplemental materials.

In total, 385 determinants were identified based on the five Consolidated Framework for Implementation Research constructs in the included studies. The qualitative studies have reported 37.1% of the determinants. The five domains did not receive similar levels of attention. A synthesis of the determinants identified in the studies is presented in Table 1. The next section will highlight the main determinants identified in the included studies based on the Consolidated Framework for Implementation Research domains.

**Table 1.** Synthesis of the determinants reported in the selected studies by the studies’ authors, by clinicians or individuals with a spinal cord injury based on the consolidated framework for implementation research domains.

Domains (%)^1^	Constructs (subconstruct)	Determinants	Studies that reported the determinants
Intervention characteristics (49.4%)	*Evidence strength and quality*	Limited evidence based (e.g., why choosing wearable powered exoskeleton over other therapies)	^17,27^
	*Relative advantage*	Offers intensive, repetitive locomotor training that may facilitate gait function recovery	^22,27,29,36,37,40,41,68,70,88^
		Patients felt more independent (e.g., compared to a harness system)	^25,29,80^
		Reduce physical strain on clinicians	^22,26–28,36,41^
		Light level of exertion using the powered exoskeleton compared to other modes of locomotion could facilitate sustained patient compliance	^31,46^
		Easier to learn than other devices	^40,80^
		Allows for easier adaptations of intervention compared to treadmill and overground gait training approaches	^27,28^
		Can enhance postural stability (including sitting balance) and increase metabolic demands through increased activation of the trunk muscles compared to the Lokomat	^37,39,74^
		It is easier to learn to use the Ekso exoskeleton, but the ReWalk offers more flexibility to the user and allows for faster walking speeds	^45^
	*Adaptability *	Many parameters of powered exoskeleton can be adjusted (e.g., step length and height) and numerous clinical strategies (e.g., level of human assistance, walking aid, session time) are possible to adjust the level of challenge	^34,42,66,70^
		Limited environments that the device could be operated on (e.g., outdoors, stairs)	^26,80^
		Can be used on different surfaces, such as on carpet and concrete.	^53^
		A formalized user training based on the level of injury may result in higher functional mobility	^5^
		Flexibility in scheduling training is essential to meet patients’ needs and increase attendance	^38,42,43^
		Use of parallel bars for training help learning to use the powered exoskeleton	^56^
		With the device being a prototype, some parameters and functionalities were new or not available during the training	^5,52^
	*Complexity*	Easy to learn to walk and to perform transfers (i.e., sit-to-stand and stand-to-sit) with the exoskeleton	^40,43,56^
		Operating is difficult and complicated for the patient, but it is getting easier with training.	^21,55^
		The learning curve to operate an exoskeleton and to become independent walker is variable	^24,33,77^
		Difficulty fitting the exoskeleton on the participants	^6,29^
		Several restrictive inclusion criteria to the use of exoskeleton	^22,26,29,78^
		Difficult use for patients with severe spasticity	^66^
		Perceived difficulty to implement powered exoskeleton in current practice	^26,27,35,68^
	*Design quality & packaging*	Learning process was facilitated by auditory or vibratory feedback from powered exoskeleton	^42,59^
		Comfortable	^6,31,43,53,61–64^
		Easy to don on and don off	^31,43,46,64^
		Perceived as safe	^6,40,48,53,56,61–64,66^
		Device malfunctions	^28,34,40,42,49,52,59^
		Padding was useful to prevent skin lesion	^32,72,76^
		Large device that could feel unnatural to wear	^24,30,55^
		Skin issues related to the contact between the exoskeleton and the user	^35,40,49^
		Many participants do not respect the manufacturer's criteria for weight and height	^62^
		Devices’ engineering characteristics may influence step triggering and walking speed	^33,45^
		Can achieve a nearly natural independent walk	^57^
	*Other consequences & adverse events*	Fractures (tibia (n = 2), femur (n = 1), foot (n = 1), talus (n = 1))	^35,65,77,79^
		Falls or near-falls	^45,49,50,79^
		Skin lesion, bruising or redness on the skin	^5,32–35,41,45,48–50,53,61,67,72,73,76,77,79^
		Pain	^21,41,53,56,61,76,79^
		Swelling or edema	^34,45,61,72^
		Dizziness	^34^
		Orthostatic hypotension	^42,58^
		Upper-extremity injuries	^34,55,67,76^
		Therapist injured	^49^
		Moderate-to-high level of cognitive and physical exertion	^21,24–26,39,43,53^
		Increased spasticity or spasms	^24,41^
		Others	^21,33,38,53,79^
	*Cost*	High purchase cost	^24,53–55^
Characteristics of individuals (29.6%)	*Knowledge & beliefs about the intervention*	Perceived and measured health benefits (e.g., overall health status, reduced pain, reduced spasms, increased walking endurance, improved sitting balance, improved bowel or bladder function)	^17,21,23–25,38,43,51,53,57,58,62–65,74,77,78^
		Perceived psychosocial, emotional and/or social benefits	^5,17,21,24,25,28,39,50,77^
		Perceived limitation of exoskeletons (e.g., fear of falling, slow walking speed, inability to replace wheelchair use, spasticity limiting device use) and several risks (e.g., fall-related injuries, skin irritation, pressure ulcers)	^17,22,24,30,31,42^
		Great utility in therapy	^24^
		Need to set clear, realistic objectives and discuss expectations	^17,22,26,27,35,43,56^
		The hope of regaining the ability to walk was well present in some participants	^23,26^
	*Self-efficacy*	Training and clinical experience were seen as crucial by clinicians in their abilities to operate the device proficiently and safely	^28^
		Therapist's skills and confidence influenced users’ ability to learn to use the PE	^54^
	*Other personal attributes*	Motivated to engage in powered exoskeleton locomotor training	^21,22,30,31,43,66,69^
		Experienced strong emotions using powered exoskeleton (e.g., exhilaration, empowerment, increased self-esteem)	^23,30^
		Some personal characteristics can influence the exoskeleton skill performance (e.g., active trunk muscles, younger age at injury onset, shorter time since injury, active lifestyle prior to the lesion)	^5,45,54,68,71,75^
		Some patient's characteristics might hinder exoskeleton use (e.g., limited arm strength, un-suitable body type, noncompliant behaviors, and comprehension abilities)	^22,28^
		Feeling vulnerable while wearing the exoskeleton	^23^
Inner setting (13.5%)	*Structural characteristics*	Exoskeleton teams are created in larger institution for training, certification, continuing education	^17^
		Variability of inpatient length of stay represent a barrier for designing protocols for newly acquired spinal cord injury	^73^
	*Network and communication*	Coordination and collaboration of a multidisciplinary teams is crucial	^29,44^
		Group discussion with other physiotherapists on admissibility and protocols to develop expertise with the device	^27^
		Contact with the company/manufacturer for technical and training issues	^42,52^
	*Implementation climate*	Supportive institutional culture is a key determinant in successful implementation in clinical practice	^26^
		Logistical (e.g., transportation, schedule), social or financial issues may limit participation	^34,35,42^
		A thorough evaluation of concurrent medical conditions is crucial	^34,35^
		High physical costs (number, duration, intensity of training) for participants	^35^
		Importance to evaluate the impact on workforce (e.g.,: caseload, time)	^27,36^
	*Readiness for implementation (Available resources)*	Importance of having several trained therapists	^17,26,27,43,44^
		Time constraints on patient's schedule	^26,27,40,60,76^
		Time for therapists to be trained and to develop protocols base on their clinical setting context	^27,40,47^
		Space for powered exoskeleton storage and for training sessions	^27,29,33,47^
		Lack of money to pay for travel for patient may impact participation	^44^
	*Readiness for implementation (Access to knowledge & information)*	Lack of guidelines, standardized comprehensive training or certification for the use of exoskeletons	^35,43^
		Participants with a spinal cord injury identified that having detailed information about the training process would have been helpful	^26^
	*Readiness for implementation (Leadership engagement)*	Administration support was necessary initially to acquire the powered exoskeleton	^29^
Outer setting (3.9%)	*Patient needs and resources*	Lack of or difficulties with transportation to the training center	^6,29,33,36,38,42,60^
		Patient demand for exoskeletons motivates purchase and use by facilities	^17^
	*External policies and incentives*	Only a few powered exoskeletons are approved by Food and Drug Administration	^5,24^
		Some patients sought out facilities for physical therapy services because they offered use of powered exoskeleton	^17^
		Canadian public health care system may have longer lengths of stay for inpatient rehabilitation compared to other jurisdictions, which may facilitate participation in a powered exoskeleton training program	^21^
		Exoskeletons considerable media coverage may potentially influence participants’ view of the device and raising expectations	^23^
		Number of covered physical therapy visits vary depending on insurance policies	^45^
Implementation process (3.6%)	*Planning*	Importance of standardization of a training program	^32,44^
		Lack of guidance regarding the integration of exoskeletons into rehabilitation therapy services	^17,21^
		Optimal timing to introduce powered exoskeleton locomotor training in acute rehabilitation services might consider bowel and bladder routines, standing tolerance, and/or cardiovascular or pulmonary status	^21,68^
		Effective powered exoskeleton training program should include appropriate user selection, proper fitting and a steady skill progression plan	^45^
		Selection of the exoskeleton device and training parameters should be determined based on lower limb motor function user preference and motivation, comfort and skill ability, and not only the level or completeness of the injury	^45^
	*Engaging*	Numerous strategies can be implemented to overcome potential barriers (e.g., telephone pre-screening interviews to minimize the number of visits, free parking, familiarization sessions)	^42^
		Participants filmed their performance to facilitate learning	^42^

^1^ Proportion of time each domain was identified in the selected studies.

The *intervention characteristics’* domain, which corresponds to the characteristics of the gait training programs using powered exoskeletons, was the most documented with 49.4% of all determinants identified. The *relative advantage* of the innovation was highlighted in several studies, as many reported that wearable powered exoskeletons can offer intensive repetitive locomotor training to the users,^27,36,37,40,70^ and can contribute to reduce physical strain on clinicians compared to conventional gait training.^26,36^ Other studies have shown increased activation of trunk muscles when using mobile exoskeletons compared to fixed exoskeletons (e.g., the Lokomat), which may contribute to improve standing and sitting balance.^37,39,74^ About the *design quality & packaging* construct, wearable powered exoskeleton were seen as comfortable,^31,43,61^ easy to don on and don off,^31,43,46^ and safe to use.^40,61,66^ Others perceived powered exoskeleton as large device that could feel unnatural to wear.^24,30,55^ Device malfunctions and technical problems were identified as potential barriers to its use in many studies.^29,34,40,42,49,52,59^ The *adaptability* of the innovation was considered limited since the exoskeleton cannot be used in all types of environments (e.g., stairs, outdoors).^26^ The *complexity* of the device was highlighted by the several eligibility criteria for users, making it difficult for clinicians to decide whether their patients are good candidate or not for this intervention.^26,29^ In addition, operating the exoskeleton can be difficult initially for the patient.^21,55^ The learning curve to ultimately use the exoskeleton independently is variable from one individual to another.^24,33,77^ Another barrier reported by most of the studies concerns the occurrence of *adverse events and other consequences* induced by intensive training with the wearable powered exoskeletons that may affect the continuation of the training program, and include falls, fractures, skin lesions, pain, orthostatic hypotension and injuries sustained by therapists.^5,21,25,26,32–35,38,40–43,45,48–50,53,55,56,58,61,67,72,73,76,77,79^ Moreover, the training process with the powered exoskeleton was perceived as both mentally and physically demanding by some participants.^21,24–26,39,43,53^ Finally, a high purchase *cost* was identified as a barrier of accessibility to this technology.^24,53–55^ In fact, Kinnett-Hopkins et al. (2020) reported that most participants experienced exoskeleton-based training in research settings, and not in clinical settings.

The *individuals’ characteristics*, which is related to the characteristics of clinicians, patient, and others involved in the program, was the second domain most mentioned, with 29.6% of all the determinants identified. A predominance of the determinants related to the *knowledge and beliefs about the intervention* was found. In fact, several health benefits have been perceived or experienced by users (either patients or clinicians), such as improved overall health, reduced pain and spasm intensity, increased walking endurance, as well as psychosocial benefits.^5,17,21–25,29,38,39,43,50,51,53,57,58,62–65,74,77^ On the opposite, limitations and risks associated with powered exoskeleton gait training have also been individually perceived or experienced by clinicians or by patients, including a limited walking speed with the exoskeleton, falls, fear of falling, and skin irritation.^17,22,24,30,31,42^ A main determinant identified for successful use of powered exoskeleton training program was the establishment of clear and realistic objectives with the patient as well as discussing patients’ expectations toward the powered exoskeleton gait training program.^17,22,26,27,35,43,56^ As reported by the study of Ehrlich-Jones and colleagues,^22^ discussions with the patients about expectations are crucial since “Patients may see others in the device and expect similar outcomes or they may find the exoskeleton does not provide the function that they expected”. From both clinicians and spinal cord injury user's perspectives, therapists’ skills and experience with the exoskeletons influenced a proficient and safe use of the device,^29,54^ which increased sense of *self-efficacy* for both clinicians and spinal cord injury users. Finally, *other personal attributes* of individuals with a spinal cord injury may facilitate or hinder exoskeleton use. Younger age at injury onset, active lifestyle prior to the lesion, active trunk muscle, and patient motivation were identified as potential facilitators,^5,21,22,30,31,43,54,66,69,75^ whereas limited arm strength, limited comprehension abilities, and body type that does not fit in the exoskeleton represent potential limitations to the use of powered exoskeleton.^22,29^

The *inner* setting domain was represented in 13.5% of all documented determinants. *Network and communication* were identified as a crucial determinant for successful implementation of a powered exoskeleton gait training program in clinical settings. Three included articles highlighted the importance of coordination and collaboration within the multidisciplinary team,^29,44^ to ensure that patients receive the appropriate therapies to meet their rehabilitation needs.^29^ Physiotherapists perceived that working with other physiotherapist colleagues provides the opportunity to collaboratively develop protocols and discuss the appropriateness of the Ekso for each patient.^27^ Another study also noted the importance of collaborating with the patient's physician to obtain medical clearance to ensure safe participation in the powered exoskeleton gait training program.^44^ Finally, keeping contact with the exoskeleton company for assistance with technical issues or training difficulties was identified as a facilitator.^42,52^

Determinants related to the *available resources* were also frequently mentioned. One obstacle reported was the difficulty for planning training sessions due to patient's time constraints. Having several trained therapists,^17,26,27,43,44^ time dedicated for powered exoskeleton locomotor training within clinicians’ caseload,^26,27^ as well as a suitable and accessible location to store the exoskeleton^27,29,33,47^ were all identified as facilitators. Supportive *implementation climate* was also identified as a key determinant in successful implementation of powered exoskeleton program in clinical practice.^26^ Some clinicians and researchers were concerned about the *compatibility* of the powered exoskeleton technology for the training of persons with spinal cord injury, as many of them have co-morbidities that could limit or affect training. The high level of involvement required from patients is not to be neglected either, in terms of physical costs (i.e., number, duration, and intensity of training sessions),^35^ transportation and financial cost to participate in a powered exoskeleton training program, which can represent another *compatibility* issue with this program. Another barrier related to the *access to knowledge and information* was the lack of information for the management of concurrent medical conditions (e.g., osteoporosis) for exoskeleton gait training.^35^

The *outer setting* domain has been little documented in the selected articles (3.9% of all documented determinants). Nevertheless, one major barrier that emerged regarding *patient needs and resources* was the lack of or difficulty with transportation^6,33,36,38,42,60^ to have access to the powered exoskeleton gait training sessions. Regarding the *External policies and incentives* construct, inpatient rehabilitation length of stay in different health care systems may affect the number of sessions that one individual can access. In addition, differences in insurance coverage may also limit accessibility to an exoskeleton training program, depending on the number of physiotherapy visits included in one's insurance coverage.^21,45^

The *implementation process* domain was also little documented (3.6% of all documented determinants). Regarding the *planning* construct, the importance of standardized training protocols to ensure safety and proficient use of wearable exoskeleton was mentioned.^32,44^ The lack of guidelines available for the integration of exoskeleton into rehabilitation services was also identified as an important barrier to the implementation process.^17,21,26,43^ The selection of the exoskeleton device, the optimal timing to introduce powered exoskeleton as well as the selection of parameters should be based on different personal factors of the user, such as lower limb motor function, standing tolerance, cardiovascular status, user preference and motivation, rather than being based solely on the level or the severity of the injury.^21,45,68^ Finally, Read and colleagues^27^ highlighted that “the role physiotherapists play in the integration of technology and new practices is crucial, so providing physiotherapists with adequate training, time, and resources is likely a key factor in successful integration”.

## Discussion

The goal of this knowledge synthesis was to report, based on the consolidated framework for implementation research, the potential determinants of implementation of an overground powered exoskeleton locomotor training program for individuals with a spinal cord injury. Most of the reported determinants were related to the *intervention characteristics* domain. It is not surprising that the context (i.e., *inner and outer settings*) was not well documented, given that wearable powered exoskeletons are a relatively novel technology in their early stages of adoption and use in clinical settings. The scarcity of the reported determinants related to the *characteristics of individuals*, the *inner* and *outer settings*, and the *implementation process* may however represent a threat to a successful passage from Knowledge to Action.^18^

Within the *intervention characteristics* domain, the overground locomotor training using a powered exoskeleton seems to present a *relative advantage* over other technologies or intervention. Their *design quality and packaging* were described as either a facilitator or an obstacle, which could be influenced by the type of exoskeleton used in the studies. The *complexity* of the technology, as well as the numerous *adverse events and other consequences* caused by training with the exoskeleton were described as important barriers to its clinical use. The complexity of an intervention is known to significantly influence the level of effort required for implementation, especially when it requires more human resources, as it is indeed the case with wearable powered exoskeleton.^81^ Another barrier identified by stakeholders is the high *cost* for purchase and maintenance of exoskeleton. However, it is crucial to evaluate the technology cost-effectiveness, and not only consider its purchase cost, since a technology may appear to be expensive at the implementation stage, but saves money on the long term. Pinto and colleagues^82^ examined relevant economic factors for exoskeleton locomotor training following a spinal cord injury and they estimated that providing powered exoskeleton overground training for 10% of locomotor training sessions results in a decreased hospital costs between $1114 to $4784 annually. Further economic studies are needed to corroborate these estimates.

Within the *inner setting* domain, the *available resources* construct was identified as a key factor in successful implementation of powered exoskeleton in clinical practice. This includes providing therapists with adequate training, time, and resources. Read and colleagues^27^ reported that physiotherapists’ adoption of technology is dependent to some extent on the willingness of their organization to support them with additional resources. In addition, some therapists in stroke rehabilitation were reticent to engage with powered exoskeleton due to the time and effort required to learn how to use the device,^83^ which again highlighted the importance of supportive institutional culture to promote implementation. Coordination and collaboration of a multidisciplinary team has been reported as an important determinant of implementation, especially at early stages of recovery following a spinal cord injury. In stroke rehabilitation using powered exoskeleton, multidisciplinary coordination between physical rehabilitation unit along with other hospital disciplines was also identified as a key determinant.^84^

Limited determinants have been identified regarding the *characteristics of individuals*. Nonetheless, a key element mentioned in several studies was the need to establish clear and realistic objectives for the use of powered exoskeleton in gait training, as well as the importance of discussing expectations with patients. Patient expectations can be managed through education, a well-informed selection process, as well as through constant re-assessment of the process,^26^ as expectations can change over time.^17^ Multidisciplinary teamwork can also be a facilitator for the management of patients’ expectations, by involving a psychologist, neuropsychologist or social worker in the discussion with the patient about his expectations towards this technology.

The *outer setting* and the *implementation process* domains were very little documented. In implementation research in general, *outer setting* tends to receive less attention compared to the others determinants.^85,86^ A systematic review concluded that context has been an inconsistently defined and applied concept throughout studies, limiting its operationalization in research and practice. A well-defined and consensual definition of this concept would help to better understand its influence on the effectiveness and reach of complex interventions.^87^ Although we have very little information on the interaction between the determinants of the five domains of the consolidated framework for implementation research, two studies have underlined that adoption of powered exoskeleton technologies does not depend solely on their effectiveness, but is rather influenced by the complex interactions between the technology characteristics, the attitudes of the various stakeholders involved, and the organization of the healthcare setting.^12,29^

Although several determinants were identified in this systematic review, future research is needed to explore more thoroughly determinants from the context (*inner and outer settings*) and from the *implementation process*. A better comprehension of the interactions between all determinants may help implementing these complex technologies, which may contribute to increase stakeholders’ acceptance and uptake of powered exoskeletons in clinical practice. Some implementation strategies can be suggested, such as developing users’ eligibility checklist, guidelines for providing training, decision support to pursue and progress powered exoskeleton gait training, as well as establishing organizational strategies in terms of resources.

Some limitations of this review should be acknowledged. First, data extraction was not completed by two independent reviewers. Another limitation of our study is that the identified determinants were not analyzed separately for different users (e.g., patients, clinicians). Finally, methodological quality assessment of the included studies was not performed due to the variety of study designs and scope of the different publications. A methodological quality assessment would not have influenced the interpretation of the results at this stage.

**Clinical messages**

- Wearable powered exoskeleton training is a complex intervention that might be challenging to implement in clinical settings. Such an implementation requires a good knowledge of the characteristics of the exoskeleton (e.g., design, functionalities) and demands adequate training for clinicians to administer safely a training program using this technology.
- For a successful implementation, key organizational elements should be considered by the healthcare setting, such as the availability of resources (e.g., time, having several trained therapists, space) and maintaining a good communication between the multidisciplinary team members.
- Management of patient's expectations and hopes toward the powered exoskeleton training program should be addressed, ideally by a multidisciplinary team, before starting the program and throughout the process to have a realistic and shared vision of the therapeutic objectives.

## Supplemental Material

sj-docx-1-cre-10.1177_02692155231164092 - Supplemental material for A Systematic Review of the Determinants of Implementation of a Locomotor Training Program Using a Powered Exoskeleton for Individuals with a Spinal Cord Injury Click here for additional data file. Supplemental material, sj-docx-1-cre-10.1177_02692155231164092 for A Systematic Review of the Determinants of Implementation of a Locomotor Training Program Using a Powered Exoskeleton for Individuals with a Spinal Cord Injury by Caroline Charette, Julien Déry, Andreanne K. Blanchette, Céline Faure, Fran?s Routhier, Laurent J. Bouyer, and Marie-Eve Lamontagne in Clinical Rehabilitation

## Appendix 1. Detailed search strategies

**Example of search strategy in MEDLINE/Ovid database**

**Table:**

**#**	**Search**
**1**	(exoskelet* or exo skelet* or ((powered or robot*) adj3 (orthos* or training)) or pgo or wearable robot* or rex or H2 or atlas or robin).ti,ab. or robotic*.ti.
**2**	Exoskeleton Device/ or Robotics/
**3**	1 or 2
**4**	(walking or walk or gait or gaits or locomotor or locomotion or ambulation or rehabilitation or mobility or overground or over ground).ti,ab.
**5**	Exercise Therapy/ or Locomotion/ or Walking/ or Gait/ or Rehabilitation/
**6**	4 or 5
**7**	(rewalk* or Ekso or elegs or mindwalker* or Wearable Power Assist Locomotor or WPAL or mina or indego or ARGO or RGO or IRGO or (reciprocat* adj3 orthos*) or arke or ExoAtlet or bionic leg* or keeogo or kinesis or tibion or Vanderbilt or ABLE human motion or Walking Assistance Device* or X1 Robotic Exoskeleton* or HULC or Human Universal Load Carrier or walktrainer* or hybrid assistive limb* or HAL).ti,ab.
**8**	(((robot* or bionic* or powered or automated) adj4 (walk* or gait* or locomot* or ambulat*)) or ragt).ti,ab.
**9**	7 or 8
**10**	(((spinal cord or spinal column) adj3 (injur* or trauma* or transection* or lacerat* or contusion* or damag* or lesion*)) or SCI or ((traumatic or posttraumatic) adj2 myelopath*)).ti,ab.
**11**	Spinal Cord Injuries/
**12**	10 or 11
**13**	**((3 and 6) or 9) and 12**
**14**	(exoskelet* or exo skelet* or ((powered or robot*) adj3 (orthos* or training)) or pgo or wearable robot* or rewalk* or Ekso or elegs or mindwalker* or Wearable Power Assist Locomotor or WPAL or mina or indego or ARGO or RGO or IRGO or (reciprocat* adj3 orthos*) or arke or ExoAtlet or bionic leg* or keeogo or kinesis or tibion or Vanderbilt or ABLE human motion or Walking Assistance Device* or X1 Robotic Exoskeleton* or HULC or Human Universal Load Carrier or walktrainer* or hybrid assistive limb* or HAL).ti,ab.
**15**	(((robot* or bionic* or powered or automated) adj4 (walk* or gait* or locomot* or ambulat*)) or ragt).ti,ab.
**16**	Exoskeleton Device/
**17**	14 or 15 or 16
**18**	(((spinal cord or spinal column) adj3 (injur* or trauma* or transection* or lacerat* or contusion* or damag* or lesion*)) or SCI or ((traumatic or posttraumatic) adj2 myelopath*)).ti,ab.
**19**	(wheelchair* or wheel chair* or paraplegi* or tetraplegi* or quadriplegi* or multiple sclerosis or stroke or ((physical* or mobility or ambulat* or walking) adj2 (disorder* or incapacit* or impair* or limit* or problem* or difficult* or deficien* or disab* or challeng* or issue*)) or disabled or disabilit* or handicap*).ti,ab.
**20**	(rehabilitation or neurorehabilitation or physiotherap* or physical therap* or exercise therap*).ti,ab.
**21**	Spinal Cord Injuries/ or Multiple Sclerosis/ or Stroke/ or Stroke Rehabilitation/ or Disabled Persons/ or Wheelchairs/ or Rehabilitation/ or Neurological Rehabilitation/ or Exercise Therapy/ or Physical Therapy Specialty/ or Physical Therapy Modalities/
**22**	18 or 19 or 20 or 21
**23**	(barrier or barriers or obstacle or obstacles or difficulty or difficulties or challenge or challenges or pitfall* or shortcoming* or facilitat* or opportunit* or experience or experiences or perspective* or survey* or view or views or opinion or opinion* or expectation* or perception* or acceptance or appraisal* or implementation or implementing or adoption or adopting or integration or integrating or state of the art or feasib* or usability or pros or cons or advantage* or disadvantage* or drawback* or satisfaction or preference*).ti. or (barrier or barriers or obstacle or obstacles or difficulty or difficulties or challenge or challenges or pitfall* or shortcoming* or facilitator* or opportunit* or experience or experiences or perspective* or survey* or view or views or opinion or opinion* or expectation* or perception* or acceptance or appraisal* or implementation or implementing or adoption or adopting or integration or integrating or state of the art or feasibilit* or usability or pros or cons or advantage* or disadvantage* or drawback* or satisfaction or preference*).ab./freq = 2
**24**	"Surveys and Questionnaires"/ or Feasibility Studies/ or exp Patient Satisfaction/
**25**	23 or 24
**26**	**17 and 22 and 25**
**27**	**13 or 26**
**28**	27 not (mouse or mice or rat or rats or rodent* or rabbit or rabbits).ti.
**29**	28 not ((treadmill* or lokomat) not (overground or over ground)).ti.
**30**	29 not ((upper limb* or upper extremit* or hand or hands or arm or arms or forearm* or shoulder or shoulders or elbow or elbows or wrist or wrists) not (lower limb* or lower extremit* or leg or legs or foot or feet)).ti.
**31**	limit 30 to (english or french)
**Example of search strategy in Web of science database (Science Citation Index Expanded, Social Sciences Citation Index, Emerging Sources Citation Index)**	
**#**	**Search**
**1**	TS = (exoskelet* or “exo skelet*” or ((“powered” or robot*) NEAR/2 (orthos* or “training”)) or “pgo” or “wearable robot*” or “rex” or “H2” or “atlas” or “robin”) OR TI = robotic*
**2**	TS = (“walking” or “walk” or “gait” or “gaits” or “locomotor” or “locomotion” or “ambulation” or “rehabilitation” or “mobility” or “overground” or “over ground”)
**3**	TS = (rewalk* or “Ekso” or “elegs” or mindwalker* or “Wearable Power Assist Locomotor” or “WPAL” or “mina” or “indego” or “ARGO” or “RGO” or “IRGO” or (reciprocat* NEAR/2 orthos*) or “arke” or “ExoAtlet” or “bionic leg*” or “keeogo” or “kinesis” or “tibion” or “Vanderbilt” or “ABLE human motion” or “Walking Assistance Device*” or “X1 Robotic Exoskeleton*” or “HULC” or “Human Universal Load Carrier” or walktrainer* or “hybrid assistive limb*” or “HAL”)
**4**	TS = (((robot* or bionic* or “powered” or “automated”) NEAR/3 (walk* or gait* or locomot* or ambulat*)) or “ragt”)
**5**	#3 or #4
**6**	TS = (((“spinal cord” or “spinal column”) NEAR/2 (injur* or trauma* or transection* or lacerat* or contusion* or damag* or lesion*)) or “SCI” or ((“traumatic” or “posttraumatic”) NEAR/1 myelopath*))
**7**	**((#1 and #2) or #5) and #6**
**8**	TS = (exoskelet* or “exo skelet*” or ((“powered” or robot*) NEAR/2 (orthos* or “training”)) or “pgo” or “wearable robot*” or rewalk* or “Ekso” or “elegs” or mindwalker* or “Wearable Power Assist Locomotor” or “WPAL” or “mina” or “indego” or “ARGO” or “RGO” or “IRGO” or (reciprocat* NEAR/2 orthos*) or “arke” or “ExoAtlet” or “bionic leg*” or “keeogo” or “kinesis” or “tibion” or “Vanderbilt” or “ABLE human motion” or “Walking Assistance Device*” or “X1 Robotic Exoskeleton*” or “HULC” or “Human Universal Load Carrier” or walktrainer* or “hybrid assistive limb*” or “HAL”)
**9**	TS = (((robot* or bionic* or “powered” or “automated”) NEAR/3 (walk* or gait* or locomot* or ambulat*)) or “ragt”)
**10**	#8 or #9
**11**	TS = (((“spinal cord” or “spinal column”) NEAR/2 (injur* or trauma* or transection* or lacerat* or contusion* or damag* or lesion*)) or “SCI” or ((“traumatic” or “posttraumatic”) NEAR/1 myelopath*))
**12**	TS = (wheelchair* or “wheel chair*” or paraplegi* or tetraplegi* or quadriplegi* or “multiple sclerosis” or “stroke” or ((physical* or “mobility” or ambulat* or “walking”) NEAR/1 (disorder* or incapacit* or impair* or limit* or problem* or difficult* or deficien* or disab* or challeng* or issue*)) or “disabled” or disabilit* or handicap*)
**13**	TS = (“rehabilitation” or “neurorehabilitation” or physiotherap* or “physical therap*” or “exercise therap*”)
**14**	#11 or #12 or #13
**15**	TI = (“barrier” or “barriers” or “obstacle” or “obstacles” or “difficulty” or “difficulties” or “challenge” or “challenges” or pitfall* or shortcoming* or facilitat* or opportunit* or “experience” or “experiences” or perspective* or survey* or “view” or “views” or “opinion” or opinion* or expectation* or perception* or “acceptance” or appraisal* or “implementation” or “implementing” or “adoption” or “adopting” or “integration” or “integrating” or “state of the art” or feasib* or “usability” or “pros” or “cons” or advantage* or disadvantage* or drawback* or “satisfaction” or preference*)
**16**	**#10 and #14 and #15**
**17**	**#7 or #16**
**18**	#17 not TI = (“mouse” or “mice” or “rat” or “rats” or “rodent*” or “rabbit” or “rabbits”)
**19**	#18 not TI = ((“treadmill*” or “lokomat”) not (“overground” or “over ground”))
**20**	#19 not TI = ((“upper limb*” or “upper extremit*” or “hand” or “hands” or “arm” or “arms” or “forearm*” or “shoulder” or “shoulders” or “elbow” or “elbows” or “wrist” or “wrists”) not (“lower limb*” or “lower extremit*” or “leg” or “legs” or “foot” or “feet”))
**21**	#20 Restrict results by languages: English, French
**Example of search strategy in CINAHL(EBSCO)**	
**#**	**Search**
**1**	TI (exoskelet* or “exo skelet*” or ((powered or robot*) N2 (orthos* or training)) or pgo or “wearable robot*” or rex or H2 or atlas or robin or robotic*) OR AB (exoskelet* or “exo skelet*” or ((powered or robot*) N2 (orthos* or training)) or pgo or “wearable robot*” or rex or H2 or atlas or robin)
**2**	MH (Exoskeleton Devices or Robotics)
**3**	S1 or S2
**4**	TI (walking or walk or gait or gaits or locomotor or locomotion or ambulation or rehabilitation or mobility or overground or “over ground”) OR AB (walking or walk or gait or gaits or locomotor or locomotion or ambulation or rehabilitation or mobility or overground or “over ground”)
**5**	MH (Therapeutic Exercise or Locomotion or Walking or Gait or Step or Rehabilitation)
**6**	S4 or S5
**7**	TI (rewalk* or Ekso or elegs or mindwalker* or “Wearable Power Assist Locomotor” or WPAL or mina or indego or ARGO or RGO or IRGO or (reciprocat* N2 orthos*) or arke or ExoAtlet or “bionic leg*” or keeogo or kinesis or tibion or Vanderbilt or “ABLE human motion” or “Walking Assistance Device*” or “X1 Robotic Exoskeleton*” or HULC or “Human Universal Load Carrier” or walktrainer* or “hybrid assistive limb*” or HAL) OR AB (rewalk* or Ekso or elegs or mindwalker* or “Wearable Power Assist Locomotor” or WPAL or mina or indego or ARGO or RGO or IRGO or (reciprocat* N2 orthos*) or arke or ExoAtlet or “bionic leg*” or keeogo or kinesis or tibion or Vanderbilt or “ABLE human motion” or “Walking Assistance Device*” or “X1 Robotic Exoskeleton*” or HULC or “Human Universal Load Carrier” or walktrainer* or “hybrid assistive limb*” or HAL)
**8**	TI (((robot* or bionic* or powered or automated) N3 (walk* or gait* or locomot* or ambulat*)) or ragt) OR AB (((robot* or bionic* or powered or automated) N3 (walk* or gait* or locomot* or ambulat*)) or ragt)
**9**	S7 or S8
**10**	TI (((“spinal cord” or “spinal column”) N3 (injur* or trauma* or transection* or lacerat* or contusion* or damag* or lesion*)) or SCI or ((traumatic or posttraumatic) N1 myelopath*)) OR AB (((“spinal cord” or “spinal column”) N3 (injur* or trauma* or transection* or lacerat* or contusion* or damag* or lesion*)) or SCI or ((traumatic or posttraumatic) N1 myelopath*))
**11**	MH (Spinal Cord Injuries)
**12**	S10 or S11
**13**	**((S3 and S6) or S9) and S12**
**14**	TI (exoskelet* or “exo skelet*” or ((powered or robot*) N2 (orthos* or training)) or pgo or “wearable robot*” or rewalk* or Ekso or elegs or mindwalker* or “Wearable Power Assist Locomotor” or WPAL or mina or indego or ARGO or RGO or IRGO or (reciprocat* N2 orthos*) or arke or ExoAtlet or “bionic leg*” or keeogo or kinesis or tibion or Vanderbilt or “ABLE human motion” or “Walking Assistance Device*” or “X1 Robotic Exoskeleton*” or HULC or “Human Universal Load Carrier” or walktrainer* or “hybrid assistive limb*” or HAL) OR AB (exoskelet* or “exo skelet*” or ((powered or robot*) N2 (orthos* or training)) or pgo or “wearable robot*” or rewalk* or Ekso or elegs or mindwalker* or “Wearable Power Assist Locomotor” or WPAL or mina or indego or ARGO or RGO or IRGO or (reciprocat* N2 orthos*) or arke or ExoAtlet or “bionic leg*” or keeogo or kinesis or tibion or Vanderbilt or “ABLE human motion” or “Walking Assistance Device*” or “X1 Robotic Exoskeleton*” or HULC or “Human Universal Load Carrier” or walktrainer* or “hybrid assistive limb*” or HAL)
**15**	TI (((robot* or bionic* or powered or automated) N3 (walk* or gait* or locomot* or ambulat*)) or ragt) OR AB (((robot* or bionic* or powered or automated) N3 (walk* or gait* or locomot* or ambulat*)) or ragt)
**16**	MH (Exoskeleton Devices)
**17**	S14 or S15 or S16
**18**	TI (((“spinal cord” or “spinal column”) N3 (injur* or trauma* or transection* or lacerat* or contusion* or damag* or lesion*)) or SCI or ((traumatic or posttraumatic) N1 myelopath*)) OR AB (((“spinal cord” or “spinal column”) N3 (injur* or trauma* or transection* or lacerat* or contusion* or damag* or lesion*)) or SCI or ((traumatic or posttraumatic) N1 myelopath*))
**19**	TI (wheelchair* or “wheel chair*” or paraplegi* or tetraplegi* or quadriplegi* or “multiple sclerosis” or stroke or ((physical* or mobility or ambulat* or walking) N1 (disorder* or incapacit* or impair* or limit* or problem* or difficult* or deficien* or disab* or challeng* or issue*)) or disabled or disabilit* or handicap*) OR AB (wheelchair* or “wheel chair*” or paraplegi* or tetraplegi* or quadriplegi* or “multiple sclerosis” or stroke or ((physical* or mobility or ambulat* or walking) N1 (disorder* or incapacit* or impair* or limit* or problem* or difficult* or deficien* or disab* or challeng* or issue*)) or disabled or disabilit* or handicap*)
**20**	TI (rehabilitation or neurorehabilitation or physiotherap* or “physical therap*” or “exercise therap*”) OR AB (rehabilitation or neurorehabilitation or physiotherap* or “physical therap*” or “exercise therap*”)
**21**	MH (Spinal Cord Injuries or Multiple Sclerosis or Stroke or Disabled or Wheelchairs or Rehabilitation or Therapeutic Exercise or Physical Therapy or Physical Therapy Practice or Physical Therapy Service)
**22**	S18 or S19 or S20 or S21
**23**	TI (barrier or barriers or obstacle or obstacles or difficulty or difficulties or challenge or challenges or pitfall* or shortcoming* or facilitat* or opportunit* or experience or experiences or perspective* or survey* or view or views or opinion or opinion* or expectation* or perception* or acceptance or appraisal* or implementation or implementing or adoption or adopting or integration or integrating or state of the art or feasib* or usability or pros or cons or advantage* or disadvantage* or drawback* or satisfaction or preference*)
**24**	MH (Surveys or Patient Satisfaction+)
**25**	S23 or S24
**26**	**S17 and S22 and S25**
**27**	**S13 or S26**
**28**	S27 not TI (mouse or mice or rat or rats or rodent* or rabbit or rabbits)
**29**	S28 not TI ((treadmill* or lokomat) not (overground or over ground))
**30**	S29 not TI ((upper limb* or upper extremit* or hand or hands or arm or arms or forearm* or shoulder or shoulders or elbow or elbows or wrist or wrists) not (lower limb* or lower extremit* or leg or legs or foot or feet))
**31**	S30 AND LA (english or french)
