# Supplementary material for: A systematic review of the determinants of implementation of a locomotor training program using a powered exoskeleton for individuals with a spinal cord injury
Source: Clin Rehabil. 2023 Apr 10;37(8):1119–38. doi: 10.1177/02692155231164092 (PMC10291847; doi:10.1177/02692155231164092)
Supplement: sj-docx-1-cre-10.1177_02692155231164092 - Supplemental material for A Systematic Review of the Determinants of Implementation of a Locomotor Training Program Using a Powered Exoskeleton for Individuals with a Spinal Cord Injury [file sj-docx-1-cre-10.1177_02692155231164092.docx]

| **Supplemental materials**  **Table 1. Characteristics of the studies included in the review** | | | | | |
| --- | --- | --- | --- | --- | --- |
| **Characteristics of the qualitative or mixed design studies included in the review** | | | | | |
| **Authors (year of publication)** | **Purpose of the study** | **Data collection and training approach (if applicable)** | **Setting** | **Participants (n)** | **Type of exoskeleton** |
| Charbonneau et al. (2021)(21) | To increase our understanding of spinal cord injury patient experience using a robotic exoskeleton in this acute post-injury period | One-on-one interviews | Acute and tertiary  neurorehabilitation units in a tertiary referral, level-1  trauma center in Canada | Motor complete or incomplete acute traumatic or nontraumatic at C6-L2 level spinal cord injury (9) | Ekso |
| Ehrlich-Jones et al. (2021)(22) | To describe the experiences of clinicians who have used robotic exoskeletons in their practice and acquire information that can guide clinical decisions and training strategies related to robotic exoskeletons | Online survey study, and 4 single-session focus groups | 3 regional rehabilitation hospitals and 1 Veteran’s Administration Medical Center | Clinicians (40, focus groups: 29) | ReWalk, Ekso, Indego, others |
| Evans et al. (2020)(23) | To document a group of participants lived experience of participating in a randomized  controlled trial of two exercise-based interventions for individuals  with spinal cord injury | Semi-structured individual interviews | Cape Town, South Africa | Traumatic motor incomplete from C1 to C8 level spinal cord injury (16) | Ekso |
| Heinemann et al. (2018)(17) | To describe clinicians’ experiences, evaluations, and training strategies using exoskeletons in rehabilitation and wellness settings | Focus groups | 4 SCI model systems (SCIMS): (1) Shirley Ryan Ability Lab (formerly the Rehabilitation Institute of Chicago), (2) Craig Hospital, (3) Shepherd Center, and (4) TIRR Memorial Hermann. | Clinicians (30) | ReWalk, Ekso, Indego |
| Kinnett-Hopkins et al. (2020)(24) | To gain insight into the experiences, perspectives, concerns, and suggestions on the use of robotic locomotor exoskeletons by civilians and veterans living with spinal cord injury | Focus group | Three regional hospitals that specialize in rehabilitation for persons with spinal cord injury | Motor complete or incomplete spinal cord injury (28) | Ekso, Indego, ReWalk |
| Manns et al. (2019)(25) | To explore the expectations and experiences of persons with spinal cord injury training with the ReWalk exoskeleton | Pre- and post-training interviews, with a follow-up interview 2 months after training.  Training parameters: 60-90 min; 4x/week; 12 weeks. | Not reported | Chronic non-progressive SCI (11) | ReWalk |
| Mortenson et al. (2020)(26) | To explore the implementation of an exoskeleton at a tertiary rehabilitation center | Longitudinal semi-structured interviews | Canadian tertiary rehabilitation center | Healthcare professionals with direct clinical experience with the exoskeleton (10) | Ekso |
| Read et al. (2020)(27) | To explore how the training and implementation of using the Ekso robotic exoskeleton with patients affects physiotherapists’ work | Interviews | A neurological rehabilitation centre in Eastern Canada | Physiotherapists (3) | Ekso |
| Swank et al. (2020a)(28) | To describe therapists’ clinical practice experiences with robotic gait training (RGT) over 3 years during inpatient rehabilitation | Survey and focus groups | Inpatient rehabilitation and outpatient locomotor clinic | Physiotherapists (10) | Ekso |
| Swank et al. (2020b)(29) | To determine the feasibility of integrating the Ekso Gait Training device into inpatient rehabilitation in a neurologic population | Surveys and focus groups  For patients, pre- and post-discharge from inpatient rehabilitation, with the completion of a survey post-discharge.  Training parameters: not reported. | Inpatient rehabilitation in a neurologic population | Physiotherapists (4) or inpatients with stroke (16) or with SCI (7) | Ekso |
| Thomassen et al. (2019)(30) | To generate new knowledge regarding user experiences of standing and walking with Ekso | Interviews | In-patient rehabilitation hospital in Norway | Persons with SCI who had trained with Ekso (3) | Ekso |
| Wolff et al. (2014)(31) | To explore perspectives of wheelchair users and healthcare professionals on reasons for use of exoskeleton technology, and the importance of a variety of device characteristics | Online survey | Not reported | Wheelchair users (354) and healthcare professionals working directly with individuals with mobility impairments (127) | NA |
| **Characteristics of the quantitative design studies included in the review** | | | | | |
| **Authors (year of publication)** | **Purpose of the study** | **Data collection and training approach (if applicable)** | **Setting** | **Participants (n)** | **Type of exoskeleton** |
| Asselin et al. (2016)(32) | To report the screening criteria, proper fitting and training procedures for use a powered exoskeleton for overground walking | Within training assessments  Training parameters: 60-90 min; 3x/week; number of sessions varied between 18-120 | James J. Peters VA Medical Center, Bronx, NY | Motor complete and incomplete spinal cord injury (12) | ReWalk |
| Asselin et al. (2021)(33) | To determine the effect of overground walking using a powered exoskeleton on soft tissue body composition in persons with spinal  cord injury | Pre- and post-training assessments  Training parameters: 60-120 min; 3x/week; 40 sessions | Medical center | Chronic spinal cord injury (8) | ReWalk |
| Bach Baunsgaard et al. (2018)(34) | To assess safety and feasibility during an 8-week training programme with the robotic exoskeletons from Ekso Bionics for person with SCI (primary objective) and to assess changes in gait function outside of the exoskeleton in the subgroup of participants who were able to walk without the exoskeleton (secondary objective) | Pre- and post-training assessments, and within training assessments  Training parameters: 3x/week, 8 weeks | 9 European SCI rehabilitation centres | Complete or incomplete spinal cord injury (C7-L2) (52) | Ekso |
| Benson et al. (2016)(35) | To assess the feasibility of conducting a well-powered trial evaluating the neurological and functional effects of using an exoskeleton in individuals with chronic spinal cord injury | Pre-, mid- and post-training assessments.  Training parameters: 2x/week; 10 weeks; 20 sessions | Specialist Spinal Cord Injuries Centre, UK | Chronic motor complete or incomplete spinal cord injury (10) | ReWalk |
| Chang et al. (2018)(36) | To investigate the feasibility of exoskeleton-assisted gait training in individuals with chronic incomplete SCI (primary objective) and to investigate the potential efficacy of exoskeleton-assisted gait training on motor and gait performance compared to conventional physical therapy gait training (secondary objective) | Pre- and post-training assessments.  Training parameters: 60 min, 5x/week; 3 weeks; 15 sessions | TIRR Memorial Hermann hospital locations in Houston, Texas | Chronic motor incomplete spinal cord injury above T12 level (4) | Ekso |
| Chisholm et al. (2017)(37) | To determine how postural control muscles of the trunk are challenged during different methods of robotic-assisted gait performance, and evaluate changes in seated balance control after gait training with robotics in people with motor-complete SCI above T6. | Three intervention phases to compare the Ekso and Lokomat methods of robotic gait training. The two groups were Ekso-Lokomat-Ekso and Lokomat-Ekso-Lokomat  Pre- and post-training assessment for each intervention, with no washout period between intervention phases  Training parameters: 10 training sessions in each intervention phase for a total of 30 sessions | Not reported | Traumatic chronic motor complete spinal cord injury (C7-T4) (3) | Ekso |
| Chun et al. (2020)(38) | To explore the effects of exoskeletal-assisted walking on bowel function in persons with spinal cord injury | Pre- and post- assessments  Training parameters: 3-4x/week; 12-14 weeks; 25 sessions | Ambulatory research facility located in a tertiary care hospital | Chronic motor-complete spinal cord injury (T1-T11) (10) | ReWalk |
| Corbianco et al. (2021)(39) | To evaluate energy cost and psychological impact during a  rehabilitation program with two different types of robotic rehabilitation systems (stationary system on a treadmill, Lokomat, and overground walking system, Ekso GT) | Within training assessments and post-training assessments  Training parameters: 60 min; 2x/week; 20 sessions | Spinal Cord Injury Unit of the University Hospital of Pisa | Chronic motor complete and incomplete spinal cord injury (7 in Ekso group and 8 in Lokomat group) | Ekso |
| Delgado et al. (2019)(40) | To examine the safety of exoskeleton-assisted walking in a spinal cord injury acute inpatient facility (primary objective) and to examine the feasibility of incorporating exoskeleton walking within daily therapy sessions (secondary objective) | Within training assessments  Training parameters: 90 min; 3x/week | spinal cord injury acute inpatient rehabilitation facility | Subacute spinal cord injury who had ambulation goals (12) | Ekso |
| Edwards et al. (2022)(41) | To demonstrate that a 12-week exoskeleton-based robotic gait training regimen can lead to a clinically meaningful improvement in independent gait speed, in community-dwelling participants with chronic incomplete spinal cord injury | Pre-, post-training, and within training assessments  Training parameters: 45 min; 3x/week; 36 sessions | Outpatient rehabilitation or research institute | Chronic incomplete spinal cord injury (9) | Ekso |
| Esquenazi et al. (2012)(5) | To assess the safety and performance of ReWalk in enabling people with paraplegia due to spinal cord injury to carry out routine ambulatory functions | Pre- and post-training assessments  Training parameters: 60-90 min; 3x/week; 8 weeks; 24 sessions | Not reported | Chronic motor complete cervical and thoracic spinal cord injury (C7-T12) | ReWalk |
| Gagnon et al. (2018)(42) | To examine the feasibility of a locomotor training program with an overground robotic exoskeleton in terms of recruitment, attendance, and drop-out rates as well as walking performance, learnability, and safety | Pre- and post-training assessments, and withing training assessments  Training parameters: 6-8 weeks; 18 sessions | Pathokinesiology Laboratory of the Centre for Interdisciplinary Research in Rehabilitation of Greater Montreal | Motor complete spinal cord injury (14) | Ekso |
| Gagnon et al. (2019)(43) | To quantify clients’ satisfaction and perception upon completion of a locomotor training program with an overground robotic exoskeleton | Post training assessment  Training parameters: 6-8 weeks; 18 sessions | Pathokinesiology Laboratory of the Centre for Interdisciplinary Research in Rehabilitation of Greater Montreal | Motor complete spinal cord injury (14) | Ekso |
| Gorgey et al. (2017)(44) | To determine whether the use of a powered exoskeleton can improve parameters of physical activity as determined by walking time, stand up time, and number of steps in persons with spinal cord injury | Pre- and post-training assessments, and within training assessments  Training parameters: 60 min; 1x/week; 10-15 weeks | Clinical rehabilitation program implemented as a part of continuum of care following discharge from rehabilitation | Men with complete and incomplete spinal cord injury (4) | Ekso |
| Hong et al. (2020)(45) | To determine the number of sessions necessary to achieve adequate exoskeletal-assisted walking skills and velocity milestones, and the percentage of participants able to achieve these skills by 12 sessions and to determine the skill progression over the course of 36 sessions. | Group 1: 12 weeks of exoskeletal-assisted walking  then crossover to 12 weeks usual activity  Group 2: 12 weeks usual activity then crossover to 12 weeks of exoskeletal-assisted walking  Assessments at 12, 24, and 36 sessions  Training parameters: 3x/week; 36 sessions | Three collaborating clinical sites (medical and rehabilitation center) | Chronic motor complete or incomplete spinal cord injury | ReWalk and Ekso |
| Juszczak et al. (2018)(46) | To explore changes in secondary health conditions that may result from using a powered exoskeleton as well as their potential impact on quality of life | Pre- and post-assessments  Training parameters: 3-4x/w; 8 weeks; 26 sessions | Five major rehabilitations institutes across the United States | Complete and incomplete spinal cord injury with a neurological level of injury of C5 and lower (45) | Indego |
| Karelis et al. (2017)(47) | To examine the effect on body composition and bone mineral density of locomotor training using a robotic exoskeleton in individuals with spinal cord injury | Pre- and post-assessments  Training parameters: 60 min; 3x/week; 6 weeks | Not reported | Non-progressive traumatic complete sensorimotor spinal cord injury (5) | Ekso |
| Kerdraon et al. (2021)(48) | To present results of the first clinical study on a newly developed robotic exoskeleton  that enables individuals with spinal cord injury to perform ambulatory functions without technical aids | Pre-, post-training, and within training assessments  Training parameters: 60 min; 3x/week; 12 sessions | Two sites specialized in spinal cord injury rehabilitation, France. | Chronic motor complete spinal cord injury ranging from T5 to T12. | Atalante |
| Khan et al. (2019)(49) | To determine the training dosage required for walking proficiency, the sensory and motor changes in the nervous system with training, and the functionality of the device in a home-like environment | Pre-, mid- and post-training assessments with a 2-3-month follow-up  Training parameters: 12 weeks | Not reported | Chronic motor complete or incomplete spinal cord injury (12) | ReWalk |
| Kim et al. (2021)(50) | To evaluate the effects of training with a newly developed powered wearable exoskeleton on functional mobility, physiological health, and quality of life in non-ambulatory spinal cord injury patients | Pre-, post-training, and within training assessments  Training parameters: 60 min; 3x/week; 30 sessions | Not reported | Chronic motor complete or incomplete spinal cord injury | Hyundai Medical Exoskeleton (H-MEX) |
| Knezevic et al. (2021)(51) | To determine the cardiometabolic demands associated with exoskeletal-assisted walking in persons with paraplegia. This study will further examine if training in the device for 60 sessions modifies cost of transport. | Pre-, post-training, and within training assessments  Training parameters: 60 min; 3x/week; 60 sessions | James J. Peters Bronx Veterans Affairs Medical Center, Center for the Medical Consequences of Spinal Cord Injury Research Center | Chronic traumatic spinal cord injury (5) | ReWalk |
| Kolakowsky et al. (2013)(52) | To evaluate the feasibility and safety using Ekso^TM^ to aid ambulation in a group of individuals with spinal cord injury who had their initial spinal cord rehabilitation. Secondarily, training effects in terms of time tolerated, distance traveled, and assistance needed while in Ekso^TM^, with progressive use were evaluated. | Pre- and within training assessments  Training parameters: 60 min; 6x/week; 6 sessions; | Spinal cord injury rehabilitation center outpatient gym | Complete spinal cord injury within 2 years of injury | Ekso |
| Koljonen et al. (2021)(53) | To examine the safety and efficacy of ambulation utilizing a semi-passive and lightweight powered exoskeleton by spinal cord injury patients. | Pre- and post-training assessments  Training parameters: 60 min; 20 sessions | US Bionics at Emeryville, CA; St. David’s Medical Center at Austin, Texas; and at The Maclehose Medical Rehabilitation Centre in  Hong Kong. | Chronic spinal cord injury (61) | Phoenix |
| Kozlowski et al. (2015)(54) | To quantify the time and effort required by persons with spinal cord injury to learn to use an exoskeleton for assisted walking | Within training assessments  Training parameters: up to 120 min; 1-2x/week; 24 sessions | Not reported | Motor complete and incomplete spinal cord injury (7) | Ekso |
| Kwon et al. (2020)(55) | To compare the energy efficiency of gait with knee-ankle-foot orthosis and robot-assisted gait and to develop a usability questionnaire to evaluate the satisfaction of walking devices in paraplegic patients with spinal cord injuries | Cross-over design (knee-ankle-foot orthosis vs ReWalk) with a 2-week wash-out period. Mid- and post-training assessments for each device.  Training parameters: 60-90 min; 4 weeks; 20 sessions | National Rehabilitation Center | Complete T1-L5 spinal cord injury  (10) | ReWalk |
| Lemaire et al. (2017)(56) | To evaluate ARKE exoskeleton training within a rehabilitation centre environment | Within training assessments and post-training assessments  Training parameters: 30 min; minimum of 4 weeks; minimum of 12 sessions | The Ottawa Hospital Rehabilitation Centre (Ottawa, Canada) | Motor complete spinal cord injury (2) | ARKE |
| Maggio et al. (2022)(57) | To evaluate the effects of intense robotic training  with the Ekso-GT on the body representation and quality of life of patients with spinal cord injury by using specific scales adapted  to assess the patients’ perception of their body representation | Pre- and post-training assessments  Training parameters: 60 min; 5x/week; 8 weeks | Robotic Neurorehabilitation  Unit of the IRCCS Centro Neurolesi Bonino-Pulejo (Messina, Italy) | Spinal cord injury (> 3 months post-injury) (21) | Ekso |
| McIntosh et al. (2020)(58) | To assess safety and feasibility for persons with acute spinal cord injury using the robotic exoskeleton | Early, middle and late training (session 2, 13, and 25) assessment, and within training assessments  Training parameters: 60 min; 3x/week; up to 25 sessions | A level-1 trauma center in Canada with both acute and tertiary inpatient SCI rehabilitation units | Complete or incomplete subacute spinal cord injury (11) | Ekso |
| Muijzer-Witteveen et al. (2018)(59) | To evaluate current experiences with wearable exoskeletons and the potential of sensory feedback from the user point of view | Post-training assessments  Training parameters: Not reported. Participants had been using the exoskeleton for at least 2 training session of more than 60 min | Sint Maartenskliniek in Nijmegen, the Netherlands | Individuals with a spinal cord injury who underwent a training program with the ReWalk (10) | ReWalk |
| Park et al. (2021)(60) | To investigate the exercise intensity of overground walking training with a robotic exoskeleton and to assess the changes in cardiorespiratory responses to robotic exoskeleton-assisted overground walking training in chronic nonambulatory patients with spinal cord injury | Pre-, mid-, post-training assessments  Training parameters: 60 min; 3x/week; 10 weeks | Research institute | Nonambulatory outpatient spinal cord injury (3) | Hyundai Medical Exoskeleton (H-MEX) |
| Platz et al. (2016)(61) | To document the device-training in terms of the achieved milestones for device use, user satisfaction, and effects on quality of life | Within training assessments and post-training assessments  Training parameters: 60 min; 5x/week; 4-5 weeks | Spinal cord injury Centre of the BDH-Klinik Greifswald | Thoracic or lumbar spinal cord injury without the ability to actively stand or walk (7) | ReWalk |
| Postol et al. (2021)(62) | To evaluate the feasibility of therapy with a free-standing exoskeleton for those with spinal cord injury, and to determine the potential health-related benefits of this intervention | Baseline, Pre-, mid-, post-training, and follow-up assessments  Training parameters: 30 min; 2x/week; 12 weeks | Not reported | Spinal cord injury (> 3 months post-injury) (3) | Rex Bionics |
| Sale et al. (2016)(63) | To evaluate the efficacy, the feasibility and the changes in the mobility and in the de-adaptations of a new rehabilitative protocol for EKSO™ | Pre- and post-training assessments  Training parameters: 45 min; 3-4x/week; 20 sessions | Not reported | Chronic motor complete or incomplete cervical and thoracic spinal cord injury (3) | Ekso |
| Sale et al. (2018)(64) | To investigate the changes in gait pattern through 3D gait analysis of subjects with spinal cord injury undergoing an adaptive training with a wearable exoskeletal device | Pre- and post-training assessments  Training parameters: 4-5x/week; 5-6 weeks; 20 sessions | Outpatient with a spinal cord injury | Chronic motor complete or incomplete C7-L2 spinal cord injury (8) | Ekso |
| Shackleton et al. (2021)(65) | To determine whether 24 weeks of Robotic Locomotor Training or Activity-based Training was sufficient time to induce bone mineral density and body composition changes in individuals with spinal cord injury | Pre- and post-training assessments  Training parameters: 60 min; 3x/week; 24 weeks | Cape Town, South Africa | Chronic motor incomplete tetraplegia (16) | Ekso |
| Shapkova et al. (2020)(66) | To evaluate the compatibility of methods and to explore the main effects of combined spinal cord electrical stimulation and exoskeleton walk training | Pre- and post-assessments and within training assessments  Training parameters: 2 weeks; 7-15 sessions | Intensive rehabilitation | Traumatic chronic spinal cord injury (35) | ExoAtlet |
| Stampacchia et al. (2020)(67) | To investigate whether persons affected by spinal cord injury can safely experience walking function using robotic exoskeletons and functional electrical stimulation | Pre-, post-training, and within training assessments  Training parameters: 50-68 min;3x/week; 20 sessions | Spinal Cord Injury Unit of the University Hospital of Pisa | Spinal cord injury (17) | Ekso |
| Swank et al. (2020)(68) | To describe the clinical characteristics and dosage associated with robotic exoskeleton gait training utilization during inpatient rehabilitation. A secondary purpose is to present functional outcomes in people with spinal cord injury and stroke after robotic exoskeleton gait training and usual care | Retrospective review of medical records. Admission and discharge assessments.  Training parameters: between 1-17 sessions. | Inpatient rehabilitation  hospital | Subacute spinal cord injury (49) | Ekso |
| Sylos-Labini et al. (2014)(69) | To report the muscle activation patterns in a sample of intact and injured subjects while walking with a robotic exoskeleton and, in particular, to quantify the level of muscle activity during assisted gait | Within training assessments  Training parameters: 1-2h; 2-3x/week; 5-8 sessions | Not reported | Healthy individuals (6) and individuals with a complete spinal cord injury from below T7 (4) | MINDWALKER |
| Tanabe et al. (2013)(70) | To address the limitations of un-motorized medial systems and motorized lateral systems by reporting on the concept, design and gait performance of a novel motorized orthosis called the WPAL (Wearable Power-Assist Locomotor) | Post-training assessments  Training parameters: 5-stage gait exercise sequence (stepping in parallel bars, gait in parallel bars, gait with treadmill, gait with walker and gait with walker and without suspension). 2-11 exercises, 60 min / exercise | Not reported | Motor complete paraplegic patients (7) | WPAL |
| Tanabe et al. (2017)(71) | To investigate gait training with a medial-type powered exoskeleton wearable power-assist locomotor (WPAL) in an individual with incomplete cervical (C5) and complete thoracic (T12) spinal cord injury | Post-training assessments and within training assessments  Training parameters: 1-2x/week; 60 sessions; 16 months | Not reported | Motor-incomplete cervical and motor-complete thoracic spinal cord injury (1) | WPAL |
| Tefertiller et al. (2018)(72) | To assess safety and mobility outcomes utilizing the Indego powered exoskeleton in indoor and outdoor walking conditions with individuals previously diagnosed with a spinal cord injury | Pre- and post-training assessments, and within training assessments  Training parameters: 3x/week; 8 weeks; 24 sessions | Outpatient clinics associated with 5 rehabilitation hospitals in the United States | Spinal cord injury with a neurological level of injury at T4 and lower (32) | Indego |
| Tsai et al. (2020)(73) | To explore the potential effects of incorporating exoskeletal-assisted walking into spinal cord injury acute inpatient rehabilitation on facilitating functional and motor recovery when compared with standard of care acute inpatient rehabilitation | Pre- and post-training assessments, and within training assessments  Training parameters: 2-7 sessions; 30 min | Spinal cord injury acute inpatient rehabilitation facility | Acute inpatient participants with spinal cord injury (30) | Ekso |
| Tsai et al. (2021)(74) | To explore the potential effect of exoskeletal-assisted walking on seated balance for persons with chronic motor complete spinal cord injury | Pre-, post-training assessments  Training parameters: 3-4 sessions (4-6 h) per week; between 7 to 90 sessions | Spinal cord injury research center | Chronic spinal cord injury (8) | ReWalk |
| van Dijsseldonk et al. (2019)(75) | To assess predictors of exoskeleton skill performance during and after exoskeleton training | Every 2 weeks assessments (total of 4 assessments)  Training parameters: 90 min; 8 weeks; 24 sessions | Sint Maartenskliniek Rehabilitation Centre in Nijmegen | Motor complete spinal cord injury (24) | ReWalk |
| van Dijsseldonk et al. (2017)(76) | To develop and test the hierarchy and reliability of a framework for measuring the progress in the ability to perform basic and advanced skills | Every 2 weeks assessments (total of 4 assessments)  Training parameters: 90 min; 8 weeks; 24 sessions | Rehabilitation Center of the Sint Maartenskliniek | Chronic motor complete spinal cord injury (12) | ReWalk |
| van Nes et al. (2022)(77) | To examine changes in quality of life after an eight-week period of robotic exoskeleton training in a homogeneous group of patients with chronic complete spinal cord injury | Pre- and post-training assessments  Training parameters: 90 min; 8 weeks; 24 sessions | Rehabilitation center of the Sint Maartenskliniek | Chronic complete spinal cord injury (T1-L1) (21) | ReWalk |
| Williams et al. (2021)(78) | To determine the feasibility of an  Ekso vs. Lokomat-assisted walking programme on lower urinary tract function in people with motor-complete spinal cord injury. Our secondary aims were to determine if the pelvic floor muscles are active during exoskeleton walking, and compare the possible effects of  2 exoskeleton training programmes on lower urinary tract function | Pre-, post-training, and within training assessments  Training parameters: 45 min walking/session; 3x/week; 12 weeks | Not reported | Chronic motor complete spinal cord injury (6) | Ekso |
| Xiang et al. (2020)(79) | To evaluate the safety and feasibility of a new rehabilitation robotic device for assisting individuals with lower extremity motor complete lesions following spinal cord injury | Pre- and post-assessments  Training parameters: 30 min; 5x/week; 2 weeks | Three hospitals in Sichuan Province, China | Complete spinal cord injury at a T6-L1 level of injury (28) | AIDER |
| Yatsuya et al. (2018)(80) | To compare the energy efficiency of Wearable Power-Assist Locomotor (WPAL) with conventional knee-ankle-foot orthoses such as Hip and Ankle Linked Orthosis or Primewalk | A-B design (knee-ankle-foot orthoses and then WPAL) with a post-training assessment for each device.  Training parameters for knee-ankle-foot orthoses: 30-60 min; 3-5 x/week; 1-3 months  Training parameters for WPAL: 30-60 min; 1-5x/week; 1-3 months | Chubu Rosai Hospital, Aichi, Japan, which is affiliated with the Japan Organization of Occupational Health and Safety. | Motor complete spinal cord injury patients with C8-L1 injuries (6) | WPAL |
| Zeilig et al. (2012)(6) | To evaluate the safety and tolerance of use of the ReWalk™ exoskeleton ambulation system in people with spinal cord injury | Pre- and post-training assessments, and within training assessments  Training parameters: Not reported. Testing was done once participants were able to walk unassisted with crutches for 100 meters. | A national spinal cord injury centre | Motor complete chronic spinal cord injury (C7-T12) (6) | ReWalk |

**Abbreviations:** min: minutes; h: hour
